# Effect of Weathering on Steel Converter Slag Used as an Oxygen Carrier

**DOI:** 10.1021/acsomega.3c04051

**Published:** 2023-11-30

**Authors:** Fredrik Hildor, Henrik Leion, Carl Linderholm

**Affiliations:** † Chemistry and Chemical Engineering, Chalmers University of Technology, 412 58 Göteborg, Sweden; ‡ Department of Space, Earth and Environment, Chalmers University of Technology, 412 96 Göteborg, Sweden

## Abstract

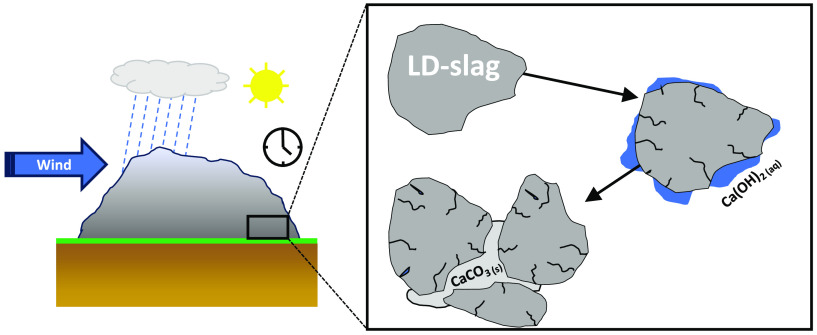

Steel converter slag, also called LD slag, is a material that has been suggested for use as a low-cost oxygen carrier for chemical looping applications. Low-cost oxygen carriers are especially relevant for the conversion of solid fuels, which may contain large amounts of reactive ashes. Ash may limit the lifetime of the bed material, which is why a high-cost oxygen carrier will likely not be competitive. Applying LD slag on an industrial scale as an oxygen carrier makes the storage properties of the material highly interesting. LD slag has been known to be affected by weathering, thus limiting the possibilities of the material to be used in construction, e.g., as fillers in concrete. In this study, pretreated LD slag for use as an oxygen carrier was weathered outdoors for roughly 1.5 years in southwest Sweden. Afterward, the particles were characterized and used in a laboratory batch fluidized bed reactor system to evaluate the effects of storage on the oxygen carrier properties. It was found that the reactivity with the fuel of the weathered LD slag was similar to that of the original sample when used in a laboratory fluidized bed. However, the physical properties were severely degraded due to weathering. Dissolved CaO formed CaCO_3_, agglomerating the top layer of the sample. The particles in the bulk of the sample were found to have decreased density and increased attrition rate. This suggests that LD slag particles for use as oxygen carriers should be stored dry to avoid weathering of the particles.

## 1 Introduction

Chemical looping techniques utilize an oxygen-carrying material to transport oxygen from one point to another. An example is chemical looping combustion (CLC) which utilizes an oxygen carrier to transport oxygen from an air reactor into a fuel reactor, where oxygen converts the fuel to carbon dioxide and water to extract the heat and energy from the fuel. The oxygen carriers are then circulated back to the air reactor. The oxygen carrier, commonly a metal oxide, is essential for the functionalities of this process. The oxygen carrier needs to chemically withstand oxidation in the air reactor and reduction in the fuel reactor. However, physical properties are just as important since the oxygen carrier will be transported between the reactors and shall withstand this stress for many cycles.^1^ The material also has to be handled before and after being used, which means storage in large piles, often outdoors.

Splitting the combustion reaction, as in CLC, mainly has two advantages:

(i) Nitrogen from the air will not dilute the flue gases generated from combustion, which is beneficial for carbon capture and storage (CCS).^1,2^ Using biomass as a fuel with CCS will also result in net negative emissions, which might be essential if our climate goals concerning CO_2_ emissions are to be reached.^3,4^

(ii) Harmful gases from the fuel are concentrated into the flue gas from the fuel without the dilution of nitrogen from the air.^5,6^ From the aspect of high-temperature corrosion of superheaters, this is an especially important property since the volatile and potent KCl is contained mainly in the flue gas of the fuel reactor, while most of the energy output from a CLC plant is extracted from the air reactor.^7^

Using biomass as fuel, such as energy crops, byproducts from agriculture, or industrial/municipal solid waste streams, to achieve negative CO_2_ emissions will introduce ash into the system. Compared to fossil fuels, the ash from biofuels is known to be chemically more reactive. This reactivity results in the ash having a higher tendency to interact with the bed material, which in turn leads to, e.g., changed reaction patterns or formation of melts due to the decreased melting point.^8,9^ Oxygen carriers are therefore expected to have a relatively short lifetime when using biofuels,^9^ and for economic reasons, low-cost oxygen carriers need to be used. Low-cost oxygen carriers include metal ores, such as the well-studied Ilmenite,^6,9^ or materials from the industry that are considered waste, such as slags.

In the Nordic region, with much steel manufacturing, one slag that has been of particular interest to be used as an oxygen carrier is steel converter slag, also called LD slag. LD slag is a byproduct of the steel manufacturing process by using a basic oxygen furnace (BOF). The slag contains mainly Ca and Fe besides Si, Mg, and Mn. Today, the demand for LD slag is low, but it is generated in large volumes resulting in high availability.^10−12^ LD slag has been evaluated as an oxygen carrier with promising results in both small scale^13,14^ and large scale.^15^ So far, LD slag has performed similar to other iron-based oxygen carriers like the well-studied iron–titanium-based ore called ilmenite.^16^ LD slag contains much higher amounts of Ca than other iron-based oxygen carriers. The high Ca content has been observed to result in lower tendencies toward agglomeration^17,18^ as well as having effects on tar cracking,^19^ sulfur interaction,^20^ and the water–gas-shift reaction that is important for gasification applications.^13^

On the market, the demand for LD slag is low partly due to its physical and chemical properties, which are also affected by the high Ca content. The high content of CaO in the slag has, for example, prohibited the extended use of the unaged material for concrete aggregates in Korea. This is due to the expansion of the unaged material weakening the cured cement.^21^ The expansion of the LD slag aggregates is related to free lime in the structure. The free lime reacts with water, forming Ca(OH)_2_, and with access to air will over time form CaCO_3_.^22^ CaO is also a leachable component from the structure that will be released from the slag over time. Experiences from using LD slag in road construction in Germany have shown that slag with up to 7 wt % free lime (CaO) can be used in unbound layers, but in asphaltic layers, slag with up to 4 wt % CaO can be used without any larger issues. However, in these studies, the MgO concentration was also low.^23^ In Sweden, the material that cannot be reused within steel manufacturing is partly used for isolation material and top-layer road constructions.^24^ The aging of LD slag, resulting in the leaching of CaO can also affect the storage properties and reactive properties when using LD slag as an oxygen carrier for chemical looping applications. CaO has been observed to be important for catalytical properties,^13^ and the fluidization properties might also change over time if the material is weakening due to weathering.

This study aims to investigate how heat-treated, sieved particles of LD slag ready for use as oxygen carriers are affected by weathering by outdoor storage. Both physical and chemical properties were evaluated for the weathered material and compared to those of a control sample. The weathered sample was stored outdoors for roughly 1.5 years and exposed to rain in southwest Sweden.

## 2 Materials and Methods

### 2.1 Material Preparation

LD slag was received from SSAB Oxelösund in Sweden. The particles were crushed, dried, and sieved to obtain the size fraction of 150–400 μm. A large batch of 38 t was produced for use in large-scale experiments,^15^ and a sample of a couple of kilos was extracted from this batch to be used in different small-scale experiments. The elemental composition of the received LD slag can be seen in Table 1. Before being used in fluidized bed experiments, the LD slag was calcined at 950 °C for 24 h in a box furnace to oxidize the sample and remove water and stored in an exicator.

**Table 1:** Elemental Composition Given in wt % of the Fresh LD Slag Sample

element	Fe	Ti	Ca	Si	Mg	Mn	V	Al	Cr	Ni
LD slag	17	0.78	32	5.6	5.9	2.6	1.5	0.76	0.33	0.002

**Figure 1:**
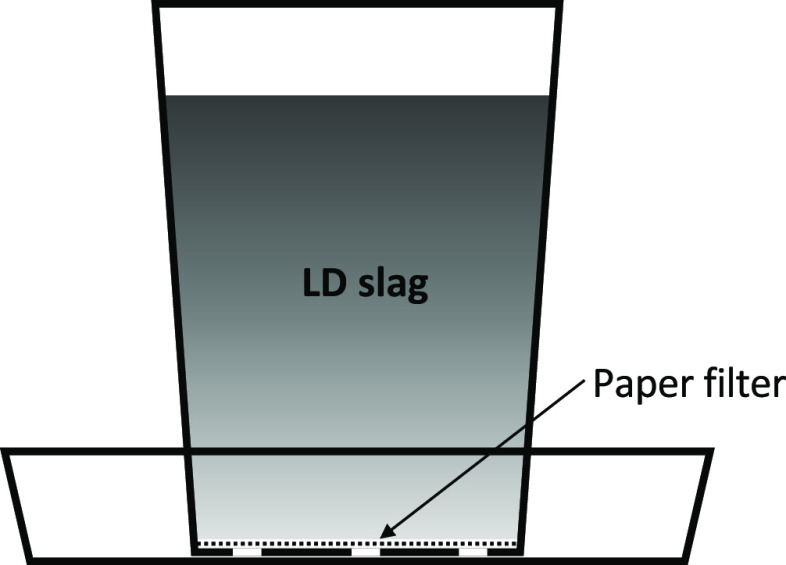
Schematic of the container where LD slag was exposed to the weather for 1.5 years.

From the same batch of materials, a sample of roughly 0.5 kg was collected for weathering. For over 1.5 years, this sample was exposed to weather and wind in the southwest part of Sweden. The sample was placed outdoors in the spring and collected in the fall the year after. In this region, the mean cumulative rain is 800–1000 mm annually, the mean temperature in summer is 16–17 °C, and in winter 0–1 °C.^25^ The sample was exposed in a perforated cup where a paper filter stopped the particles to migrate from the cup; see the schematic in Figure 1. Rain poured through the material, and the leachate was collected in the bottom collector. The leachate in the bottom collector was over drier periods evaporated and during the excess of rain drained over the edge of the collector.

### 2.2 Laboratory Fluidized Bed Reactor

**Figure 2:**
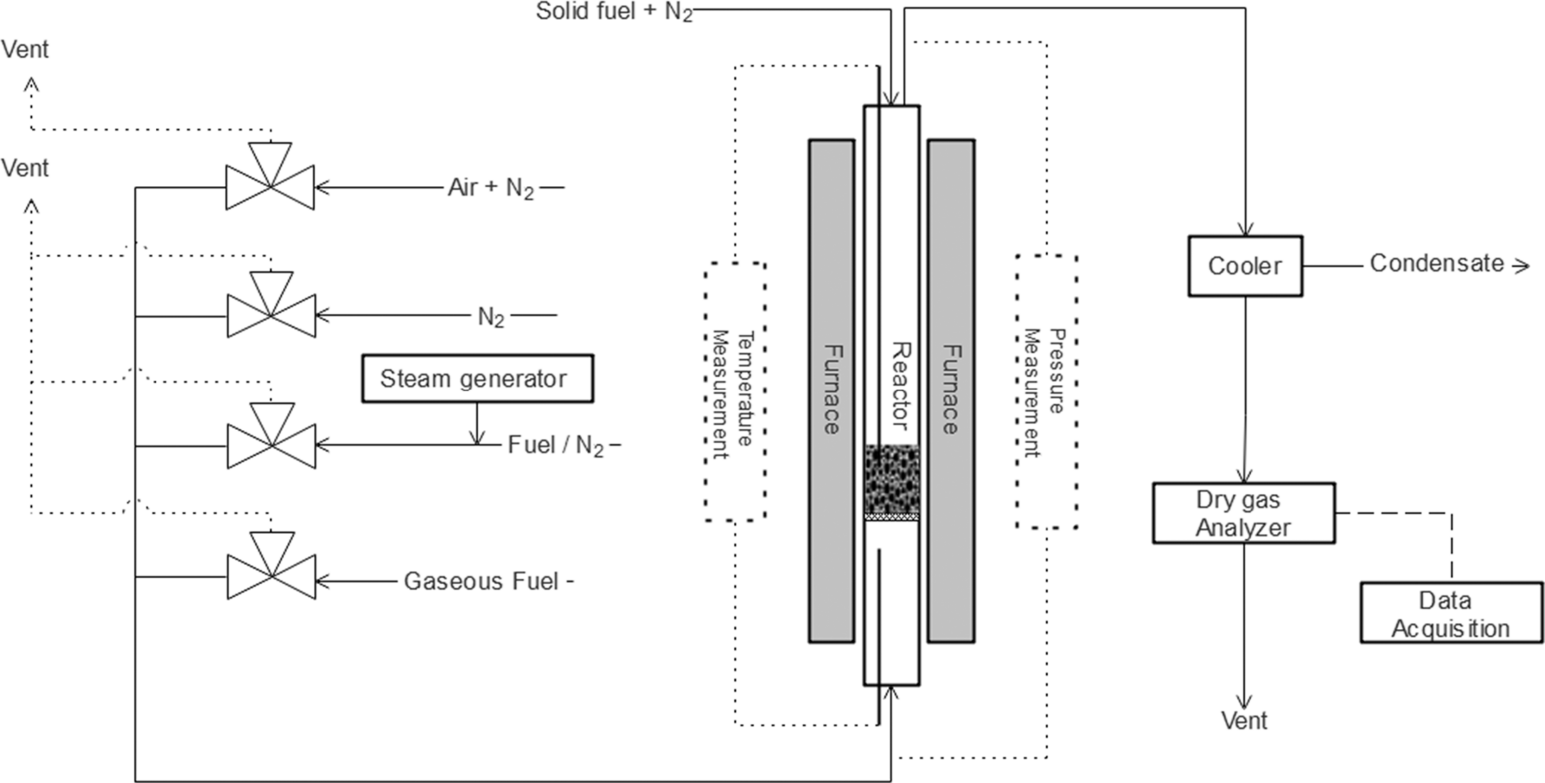
Schematic layout of the laboratory fluidized bed reactor used for reactivity experiments.

A schematic overview of the laboratory fluidized bed reactor system can be seen in Figure 2. The laboratory fluidized bed batch reactor was constructed of quartz glass with an inner diameter of 22 mm, with a porous plate placed 370 mm from the bottom, where particles were placed. The reactor was mounted inside an electrically heated furnace and was equipped with a pressure sensor (Honeywell pressure transducer, 20 Hz) to monitor the pressure fluctuations over the reactor. Two K-type thermocouples enclosed in quartz glass, one mounted in the bed and one mounted under the porous plate, were used to determine the temperature in the bed.

Ingoing gases were mixed and inserted from the bottom of the reactor. Tubing before and after the reactor was electrically heated to prevent the condensation of steam to water. Steam was generated from a CEM system (controlled evaporation mixing), and gases were mixed prior to the reactor.

After the reactor, the outgoing gases were cooled to condense and separate water. The dry gases were then analyzed using an in-line IR/UV/thermal conductivity/paramagnetic Rosemount NGA 2000 system, analyzing CO, CO_2_, CH_4_, H_2_, and O_2_. The frequency of the gas composition measurements was 1 Hz. A detailed overview of the laboratory system is presented elsewhere.^26^

#### 2.2.1 Experimental Procedure

**Figure 3:**
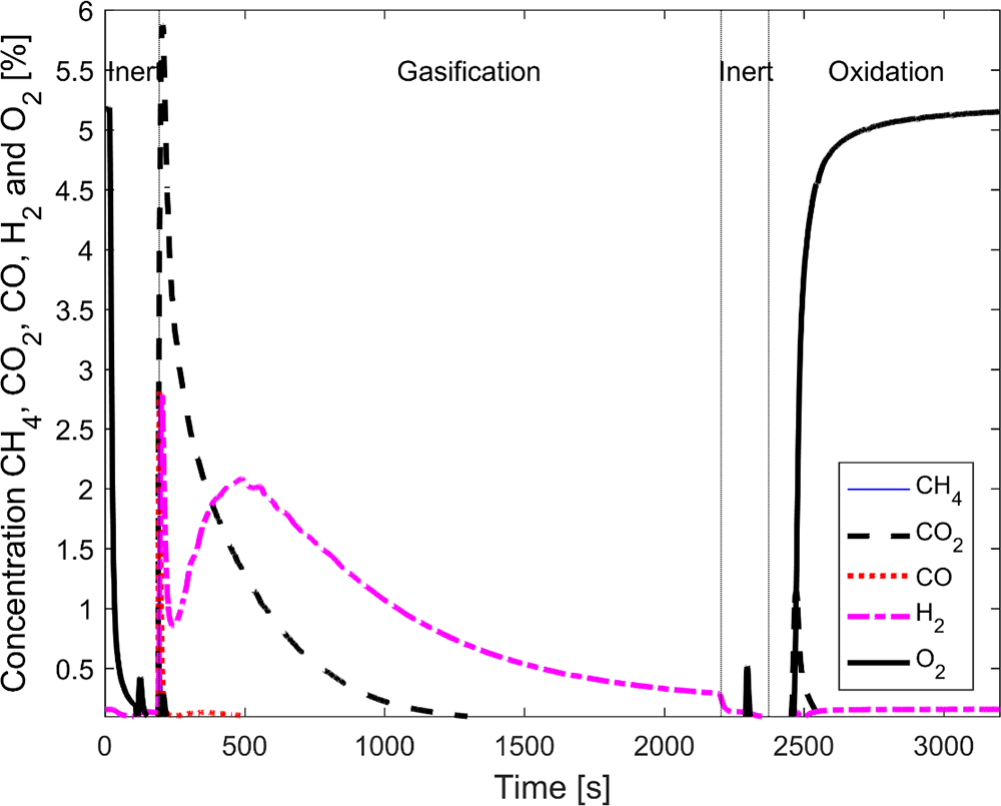
Plot of raw gas concentrations in the outlet during a cycle using solid fuel as a reducing agent when weathered LD slag was used as the oxygen carrier and the temperature was set to 870 °C. Solid fuel was introduced in the beginning of the phase called “gasification” in the figure while the bed was fluidizing with 50% steam in N_2_ to gasify the fuel.

For every experiment, a sample of 40 g of bed material was used. The atmosphere in the reactor was altered in cycles to obtain the oxidation and reduction of the oxygen carrier. One cycle is defined as an inert purge, followed by reduction, again an inert purge, and finally oxidation. These inert–reduction–inert–oxidation cycles were used wherever a solid or gaseous fuel was used in the experiments. An example of a raw data plot from one cycle using solid fuel can be seen in Figure 3. Heating of the system was performed under an oxidizing atmosphere using technical air diluted with N_2_ to obtain a flow of 1000 mL/min 5% O_2_ in N_2_. These flows were also used during oxidation until the oxygen carrier was fully oxidized, e.g., the outgoing O_2_ concentration was constant, normally 900 s. Inert purges were performed using 1000 mL/min of N_2_ for 180 s.

First, the material was activated during several cycles of reduction with 900 mL/min 50% CO in H_2_ for 20 s at 850 °C. The activation cycles were performed until stable conversion could be observed between one cycle to the next, normally 10–20 cycles.

For experiments with solid fuels, 0.2 g of char was used as fuel. This was inserted in the top of the reactor, together with 500 mL/min N_2_. The char was German wood char (Schütte) delivered already as char and was only crushed and sieved to obtain the desired size range of 180–300 μm. Fuel analysis of the char is seen in Table 2. The bed was then fluidized with 1000 mL/min of 50% steam in N_2_ from the steam generation unit. The steam/N_2_ mixture was added to the bed until the outgoing concentration of CO_2_ was below 0.1%; for experiments at 870 °C, this took around 2000 s, and at 970 °C, it took 900 s. Three different temperatures were tested, 870, 920, and 970 °C. Steam was selected as the gasification agent for the solid fuel experiments since it is commonly used in industrial applications and does not interfere with carbon measurements.

**Table 2:** Fuel Analysis of Char Used in the Experiments^a^

	dry basis					on ash samples							
	ash	S	C	H	O	Fe	Ca	Mg	Mn	Al	K	P	Si
German wood char	7.2	0.02	85.3	2.6	4.4	1	12	0.97	0.37	1	4.4	0.35	12

a Samples were converted into ash at 550 °C according to SS-EN-ISO 18122. Values are given in wt %.

#### 2.2.2 Data Evaluation

In the fluidized bed experiments, quantification of the outgoing gases was calculated as the molar flow multiplied by the concentration of the species integrated over time; see eq E1.
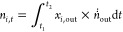
E1

The conversion of a fuel species over a cycle, γ_Fuel_, was determined by comparing the outgoing fuel species to the ingoing species (see eq E2). The molar flow for gaseous fuel experiments was calculated from the volumetric flow, assuming ideal gas.
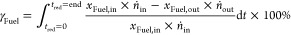
E2

The oxidation state of the oxygen carrier, ω, is defined by the mass of the oxygen carrier divided by the oxidized weight of the oxygen carrier, see eq E3. At a specific time, the mass of oxygen transferred to the fuel can be determined from the exhaust gases and ω at the time *t*_*i*_ calculated. For a fuel containing only CO, eq E4 is used.
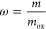
E3
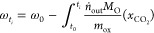
E4

To determine the oxidation level of the oxygen carrier at the end of the exposure, eq E5 was used. Here, the oxygen absorption is calculated for an oxygen carrier in comparison to that of an inert material in the same experimental setup. The difference between the O_2_ concentration is the absorbed oxygen into the oxygen carrier for one cycle. In solid fuel experiments, there are both traces of soot and some remaining char; the final O_2_ consumed by the oxygen carrier is calculated by subtracting the molar CO_2_ generated during oxidation from the total O_2_ uptake during the same period.
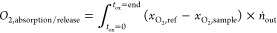
E5

Equation E5 was also used to estimate the oxygen release via CLOU (chemical looping with oxygen uncoupling) from the oxygen carrier material. This was done during the inert phase of the cycle after the sample was oxidized by comparing the oxygen carrier to a reference experiment using silica sand. The amount of oxygen released during this inert phase was then given as g O_2_/100 g oxygen carrier. For LD slag, the relative oxygen transport by CLOU is very limited compared to other oxygen carriers, but it is still measurable using this technique.^14^

For the solid fuel experiments, the total molar gas yield for each component from the gasification of the char for a cycle, or a part of a cycle, was calculated with eq E1 between time *t*_1_ to time *t*_2_. The total molar flow was calculated using eq E6. Assumptions are that the only outgoing gases of significant volume in the noncondensed gas are CO_2_, CO, H_2_, and CH_4_.
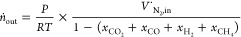
E6

The fraction of char conversion, *X*_C_, is defined as the cumulative carbon released at a certain time divided by the total carbon emitted from the converted char during the reduction period. These are defined in eqs E7 and E8. The integral in eq E8 is evaluated on the data from char insertion (*t*_1_= *t*_red, start_) until the end of the reduction when the atmosphere is changed to inert (*t*_2_ = *t*_red, end_).
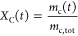
E7
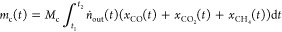
E8

The gasification rate (*r*_w_) was calculated using eq E9, where *ṁ*_c_ was the mass-based rate of conversion of carbon. The gasification rate was then normalized using eq E10. In this work, a mean value for the gasification rate was normally extracted from the gasification rate between the char conversion of 0.3 < *X*_C_ < 0.7, as has been done in earlier studies.^27^ The reason for this conversion span is that the gasification rate is more stable in this region and easier to compare to those of other experiments.
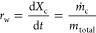
E9
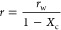
E10

The equilibrium constant *K*_eq_ for the water–gas shift (WGS) reaction (R1) is defined as in eq E11 when gases are at equilibrium. Here, *x* is defined as the incoming fraction of gas species. The temperature dependence of the equilibrium at atmospheric pressure is shown in the same equation.^28,29^ The reaction quotient *Q_i_* is defined similarly as the equilibrium and calculated for the outgoing gases from the reactor at time *i;* see eq E12. *Q_i_* is used to estimate how far the concentrations of the gases are from the equilibrium. If *Q*_*i*_ = *K*_eq_, then the gases are at equilibrium with respect to reaction R1.
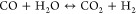
R1
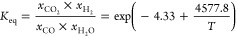
E11
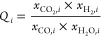
E12

### 2.3 Material Characterization

SEM-EDX (scanning electron microscopy equipped with energy-dispersive X-ray spectroscopy) was used to investigate the element interactions inside the particles. The SEM-EDX analysis was done with an FEI Quanta 200 FEG ESEM system. To expose the cross section of the particles for SEM-EDX analysis, the bed material was mounted in epoxy and polished until the particle cross section was exposed.

The crystalline phases in the bed material were determined by powder X-ray diffraction (XRD). The XRD system was a Bruker D8 Discover instrument equipped with a Cu Kα radiation source. Analysis was made between 10 and 90 2θ° with a step size of 0.02°.

It is known that LD slag contains significant amounts of CaO, Ca(OH)_2_, and CaCO_3_. These are also leachable products that can be expected to be affected by long-term outdoor storage. Quantification of CaO + Ca(OH)_2_ was provided by leaching of 1.0 g of sample in 100 mL of 10 wt % sugar solution. The sample was ground with a marble mortar and pestle and then leached for 25 min under constant stirring to provide sufficient time for CaO to react with water to form Ca(OH)_2_ and then dissolve into the water. Then, the mixture was filtered and rinsed, and the filtrate was titrated with 0.075 M H_2_SO_4_. The acid consumption was then calculated to determine the total free calcium content as CaO equivalents in the sample.^30^

The specific surface area for particles was evaluated by using BET (Brunauer–Emmett–Teller) analysis. A Micromeritics Tristar 3000 instrument operated with liquid nitrogen was used for these analyses. Before the analysis, the samples were degassed under a flow of nitrogen, 1 h at 90 °C, followed by 4–16 h at 250 °C.

Elemental analysis was performed with X-ray fluorescence (XRF) using a Panalytical Axios equipped with holders for powders. 0.5–1 g samples were pulverized using a marble mortar and pestle before being analyzed. The column with SUM indicates the estimated amount of all identified species in their stable oxides, MgO, Al_2_O_3_, SiO_2_, CaO, TiO_2_, V_2_O_5_, P_2_O_5_, MnO, and Fe_2_O_3_. If the SUM does not add up to 100, it is an indication that the elements are not present in an oxidized form or contain elements that are not detected using XRF, e.g., such as carbonates, hydroxides, and/or crystalline water.

Mechanical strength was measured using a customized jet cup attrition rig. The rate of attrition for particles was determined using a sample of 5 g in the size range of 125–180 μm. The cup in the bottom of the rig where the sample is placed has a conical inner diameter of 13/25 mm and a height of 39 mm. A nozzle with an inner diameter of 1.5 mm is placed from the side at the bottom of the cup. During operation, air is flushed into the nozzle, reaching a velocity of approximately 100 m/s. This creates a vortex, making particles swirl upward toward the settling chamber. In the settling chamber, the velocity is decreased to roughly 0.005 m/s, allowing particles with a higher terminal velocity to fall back into the cup. Particles small enough to leave the settling chamber, ideally particles <10 μm, are collected in a filter after the settling chamber. The filter is weighed every 10 min during the 1 h test to determine the attrition rate of the particles and their overall attrition trends. A closer analysis and more details regarding the determination of attrition rate and trends can be found elsewhere.^31^

Bulk density was evaluated for particles using the funnel method, according to ISO 2923–1:2008. The test was repeated three times, and the mean was calculated. Size distribution was measured by sieving with 6–7 stacked sieves, spanning from mesh size 45 to 500 μm. A sample of 20–30 g was analyzed, and an automatic vibrator was used for 20 min for the sieving.

## 3 Results and Discussion

### 3.1 Physical Appearance after Weathering

**Figure 4:**
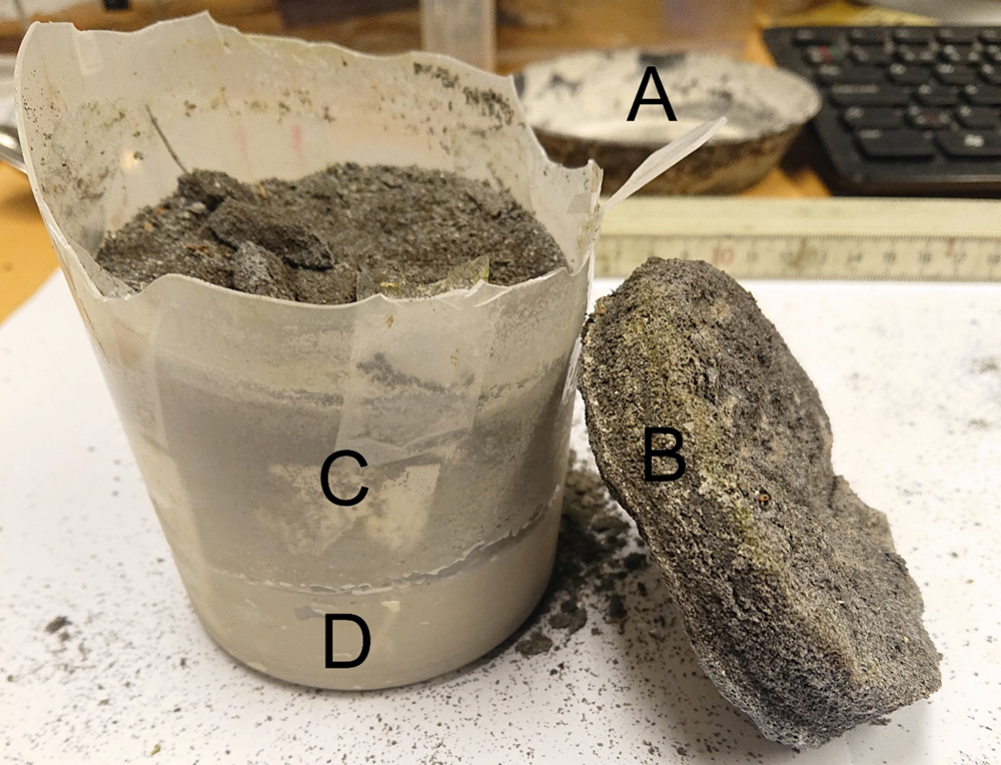
Cracked open container contains the bulk (C) of the particles and the particles below the waterline (D). The container was cracked to extract the “lid” of agglomerated particles (B), which was hard and could support its own weight. The collector with its white dried leachate is seen in the background (A).

When the weathered sample was collected after 1.5 years of unprotected outdoor weathering, it had been dry weather, and the leachate in the bottom collector had dried. A white powder was formed in the bottom collector from the leachate. The material in the main container had a very hard crust of almost 20 mm, forming a “lid” for the remaining material. Underneath this lid, called “the bulk”, the particles were not agglomerated and were easily collected. In the bottom part of the container, at level with the rim of the collector, the material was of the color of the leachate and separated from the bulk. The white leachate in the collector (A), the agglomerated particles forming a crust (B), and the container with the bulk (C) and the material below the waterline (D) can be seen in Figure 4. The “lid”, standing on the side in the figure, was very hard, and the container needed to be cut open to extract it.

**Figure 5:**
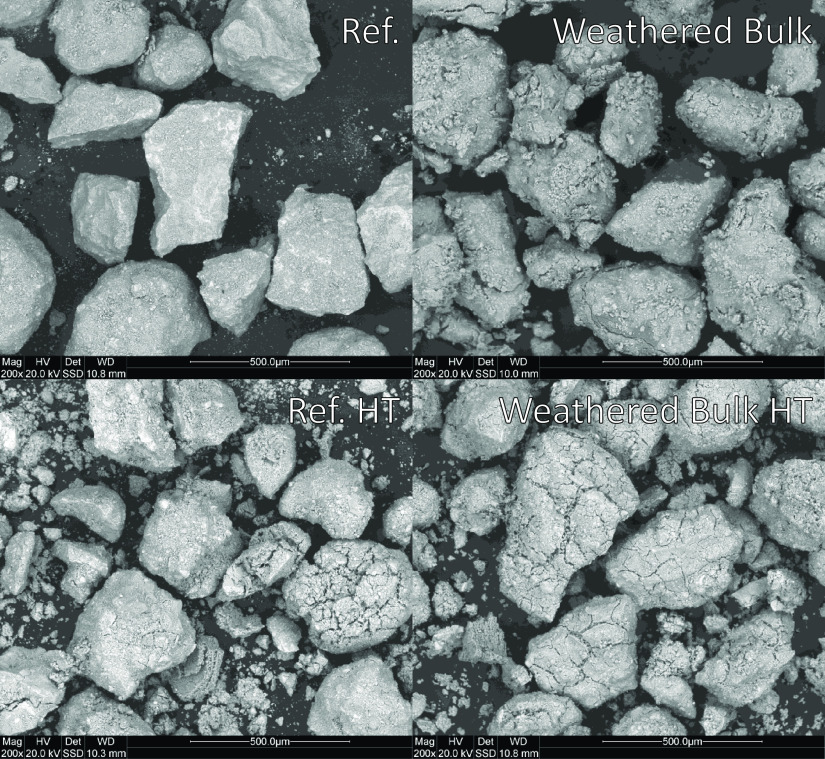
SEM images of the surface of both the reference material and weathered bulk sample. The upper row is fresh, and the bottom row is after heat treatment.

The “bulk” of the weathered sample was used in the laboratory fluidized bed batch reactor. For comparison of the material structure, the material was heat-treated in the same manner as the reference material, in a box furnace for 24 h at 950 °C. The cross sections of the reference material and the weathered material were too similar to identify any differences. However, looking at the surfaces of the materials as in Figure 5, clear differences can be seen. On the surface of the weathered material, attachments, “satellites”, can be observed. These contain calcium and are most likely easily removed by attrition. Both heat-treated samples show evidence of cracking, but cracking is more pronounced for the weathered material.

**Figure 6:**
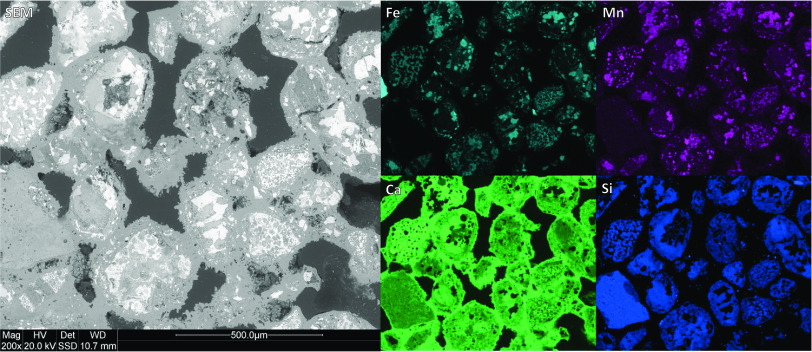
SEM-EDS of the cross section of the particles in the agglomerated “lid”. The particles were attached to each other by a Ca-containing phase, formed during weathering.

When investigating the cross section of the agglomerated particles in the “lid”, it was observed that linkages between the particles had formed, leading to agglomeration, as shown in Figure 6. These links between the particles consisted of mainly Ca with traces of other elements. The CaO + Ca(OH)_2_ content of 0.9 ± 0.3 wt %, determined by titration, was far lower than that in the bulk of the sample; see Table 4. This suggests that CaCO_3_ was formed from the interaction with CO_2_ in the surrounding air, and by that the lid is evolving downward as the diffusion of the CO_2_ penetrates the sample.

**Figure 7:**
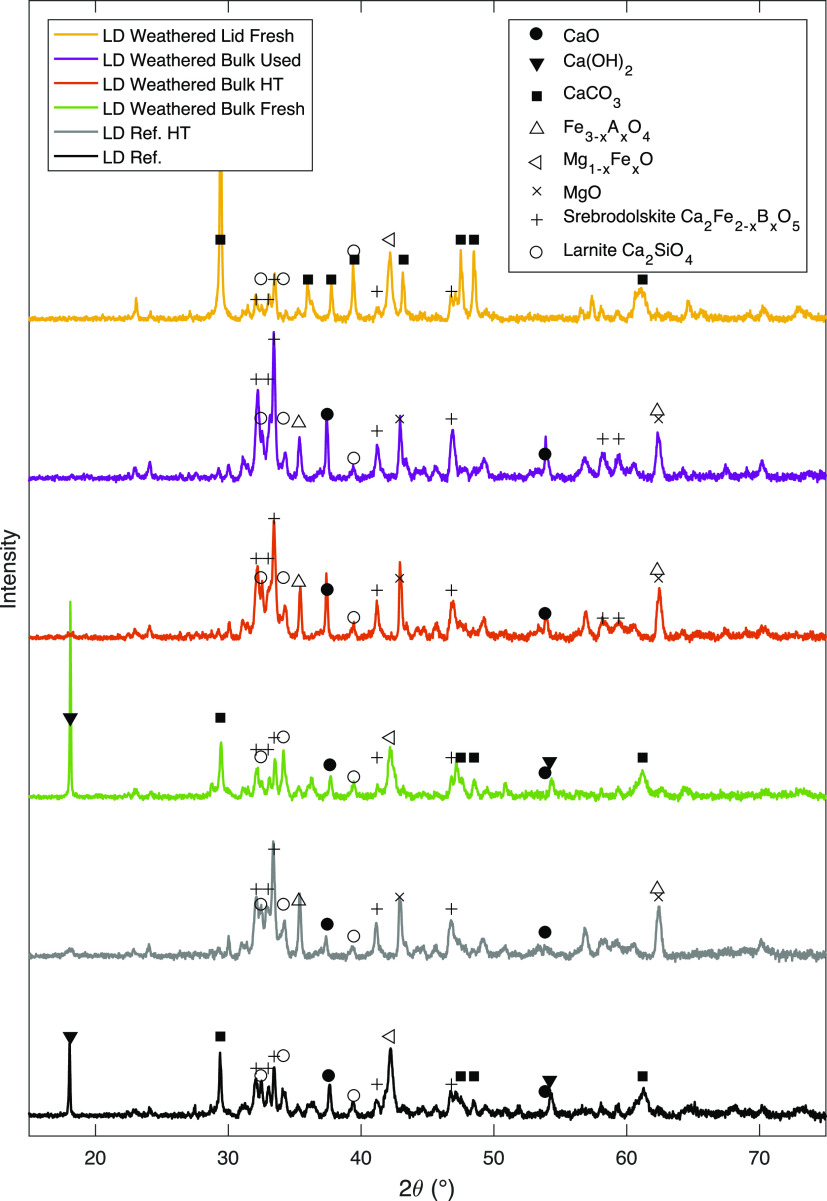
XRD diffractogram and identified phases for reference LD slag and weathered LD slag. The reference sample was both stored indoors and heat-treated (HT). For the weathered LD slag, two different fractions were analyzed: (i) “bulk” and (ii) “lid”. The bulk was analyzed as fresh, heat-treated, and after being used in the fluidized bed reactor. The “lid” of the agglomerated LD slag was evaluated without any heat treatment.

XRD diffractograms of fresh, heat-treated, and used samples are displayed in Figure 7. The identified peaks are marked with icons correlating to different phases. Black-filled markers are related to “free” calcium phases such as CaO, Ca(OH)_2_ and CaCO_3_. It can be concluded that CaCO_3_ was present in the “lid” to a significantly higher degree than in the other samples. In the fresh samples, Ca(OH)_2_ is more dominant for both the weathered bulk and the untreated fresh LD slag sample. In the heat-treated and used samples, CaO was the dominant form of these calcium species.

In Table 3 , the elemental composition from the XRF analysis is displayed. Here, it can be observed that there is only a small decrease in calcium content in the sample. It can also be observed here, as in the XRD analysis, that the elements are not present in their common oxidized form. This occurs since the sum of measured elements does not add up, indicating the presence of, e.g., hydroxides and carbonates. It can also be observed that the amount of calcium is not significantly higher below the waterline compared to that of the bulk. This suggests that the amount of leached compounds deposited below the waterline is very small compared with the material itself.

**Table 3:** Elemental Composition Given in wt-% Detected with XRF of the Reference Particles and Different Fractions of Weathered Particles^a^

sample	Mg	Al	Si	P	Ca	Ti	V	Cr	Mn	Fe	SUM
LD ref. as received	2.05	0.64	4.39	0.17	26.5	0.81	1.55	0.38	2.14	14.6	80.09
LD ref. HT	1.89	0.57	4.60	0.20	29.3	0.64	1.78	0.49	2.24	14.7	84.79
LD bulk fresh	0.81	0.33	3.91	0.17	23.4	0.60	1.50	0.38	1.93	12.8	68.88
LD bulk HT	0.99	0.39	4.24	0.19	24.1	0.60	1.54	0.39	1.94	14.0	72.74
LD lid fresh	0.67	0.29	3.69	0.18	23.6	0.57	1.44	0.36	1.85	12.5	67.45
LD below waterline fresh	0.79	0.34	3.77	0.17	23.2	0.60	1.49	0.35	1.87	12.9	68.14

a Both the reference and bulk samples were heat-treated for comparison. SUM indicates whether the amount of oxides of the quantified elements adds up to the sample weight.

**Figure 8:**
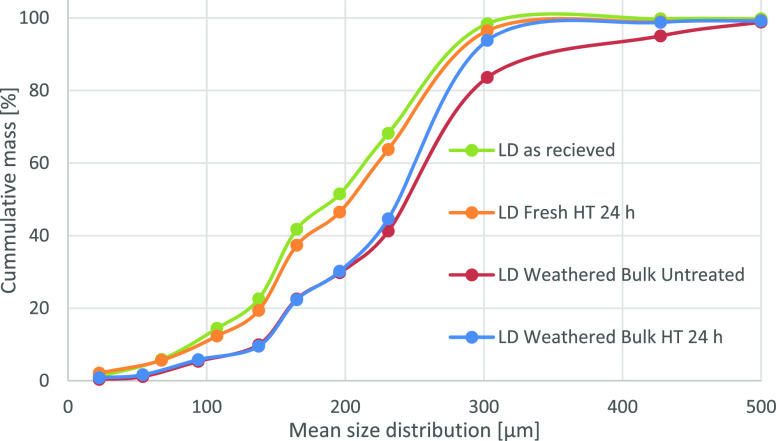
Cumulative size distribution of fresh and weathered LD slag with or without heat treatment (HT).

For reactivity experiments in the laboratory, fluidized bed particles from the “bulk” were used. These particles were heat- treated in atmospheric conditions at 950 °C for 24 h as the as-received LD slag samples. Both the untreated and heat-treated particles’ physical properties can be compared in Table 4 and Figure 8.

**Table 4:** Physical Properties of the Different Particles, before and after Heat Treatment (HT)

sample	bulk density [kg/m^3^]	BET surface area [m^2^/g]	attrition [wt %/h]	free CaO + Ca(OH)_2_ [wt %]
LD ref. as received	1560	4.4 ± 0.008	2.2	6.2 ± 0.3
LD ref. HT	1410	1.0 ± 0.005	2.2	4.0 ± 0.1
LD bulk weathered untreated	1171	10 ± 0.04	1.7	6.4 ± 0.3
LD bulk weathered HT	1162	1.4 ± 0.005	3.5	4.6 ± 0.1

From this, it can be observed that the bulk density is significantly lower for the weathered samples. Also, the heat treatment for the weathered particles resulted in the disentanglement of the particles with a size of >200 μm. Smaller particles present in the received LD slag appear from the distribution curve in Figure 8 to be removed, either washed away with the rainwater or agglomerated into larger particles or on particle surfaces. The weathered particles also had an increased attrition rate after heat treatment, suggesting a decreased physical strength compared to the particles before weathering. This could be related to the cracks formed during the heat treatment of the material, as observed on the surface SEM images displayed in Figure 5.

By themselves, these findings of changes in the physical properties suggest that LD slag treated to a specific size to be used as an oxygen carrier should not be exposed to weathering. Apparently, from the increased attrition rate, it is observed that the structural integrity of the particles is weakened by the weathering. Since the attrition rate was already high, resulting in a large amount of fines during operation in a 12 MW boiler,^15^ an increased attrition rate is not promising. Also, the ability to agglomerate due to the formation of CaCO_3_ bonding particles together will be problematic for storing outdoors.

### 3.2 Reactivity of Weathered LD Slag

From the XRD diffractograms in Figure 7, it could be observed that the oxygen-carrying spinel phase (Fe_3–*x*_A_*x*_O_3_)^14^ was observed in the heat-treated weathered sample as in the reference. From the aspect of reactivity, the effect of weathering was limited. During the final activation cycles, both the weathered and reference samples obtained 94% CO conversion when the 40 g sample was exposed to 900 mL/min 50% CO in H_2_ for 20 s. Oxygen released by CLOU was determined to be 0.3 g/kg for both the reference and weathered oxygen carrier for the last activation cycle at 850 °C. This suggests that the material changes due to weathering are limited regarding the oxygen-carrying phases.

**Figure 9:**
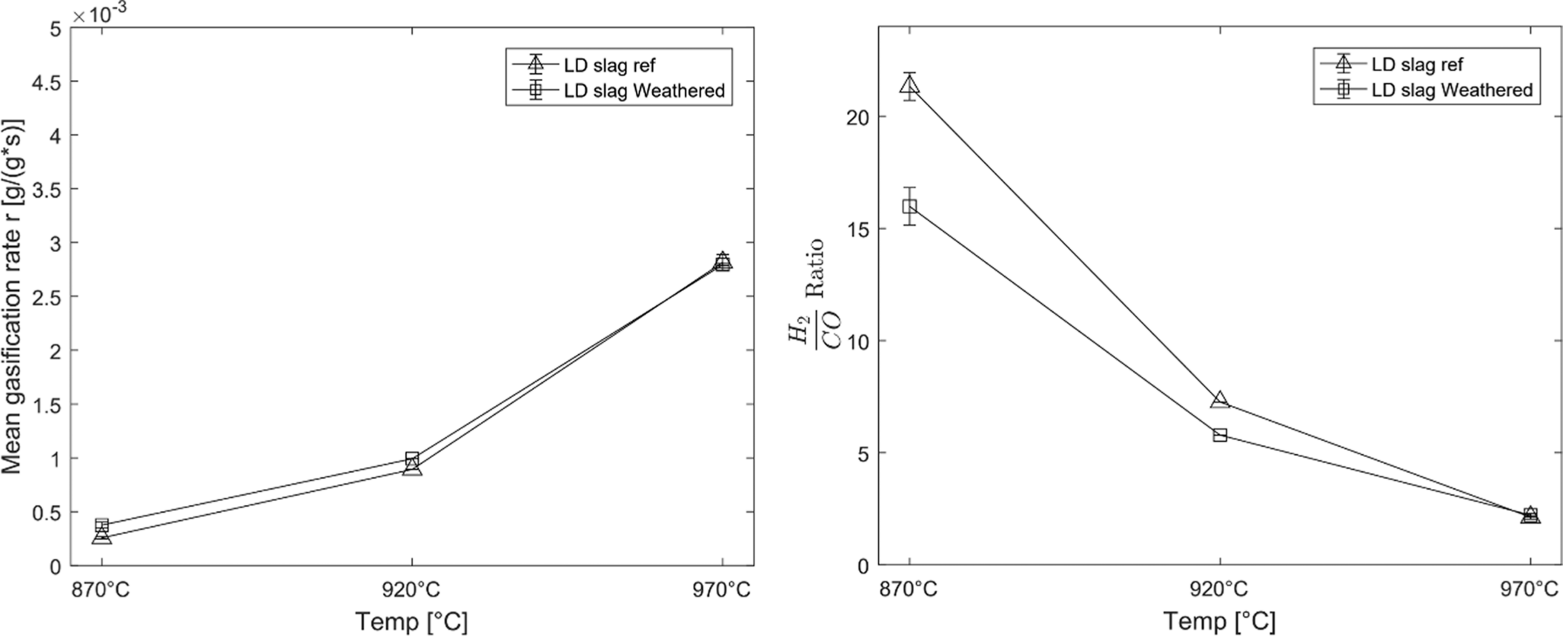
Left: mean gasification rate (mean taken from 0.3 < *X*_C_ < 0.7) for LD slag that has been weathered compared to a reference sample of LD slag at different temperatures. Right: mean H_2_/CO ratio taken from 0.3 < *X*_C_ < 0.7 with solid fuel for weathered LD slag compared to a reference sample of LD slag at different temperatures.

When experiments were performed using solid fuel, a small difference between the weathered and reference samples in gasification rate and raw gas composition could be observed at 870 °C, as seen in Figure 9. This difference disappeared when increasing the temperature. The H_2_/CO ratio of the outcoming combustible gases was almost the same but for the experiments at 870 °C where the reference sample had a higher H_2_/CO ratio. Nevertheless, the general trend is still the same compared to other bed materials when used in similar experiments with a high H_2_/CO ratio at 870 °C and decreasing while increasing the temperature. At these lower temperatures, the reaction is dominated by the WGS reaction and the reduction of H_2_O forming H_2_ and O_2_ called water splitting.^13^ The lower amount of hydrogen could also be the reason why the mean gasification rate for the weathered LD slag is higher due to the decreased hydrogen inhibition for the gasification of the char.^32^

**Figure 10:**
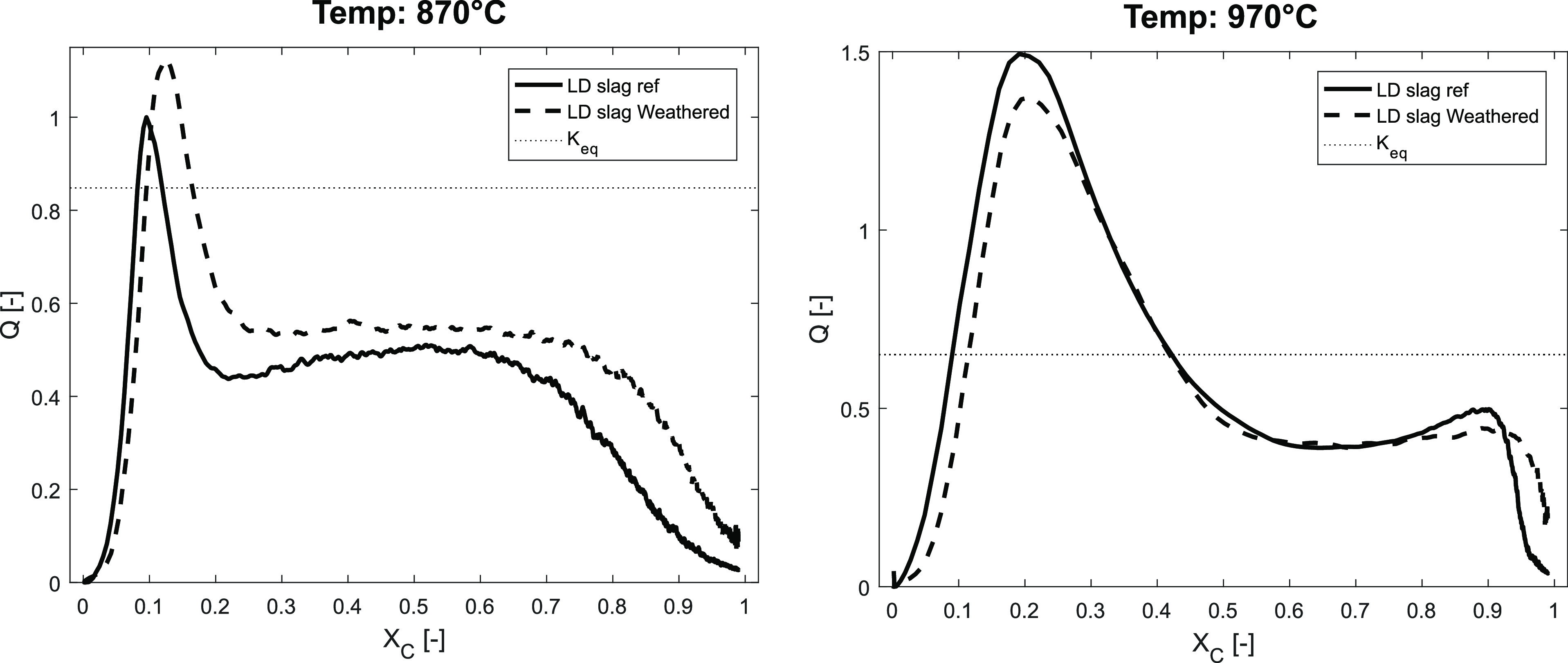
Reaction quotient *Q* for the WGS reaction as the conversion of the solid fuel propagates different temperatures to the left: 870 °C, and to the right: 970 °C. The equilibrium constant at the set temperature can be seen as a horizontal dotted line.

As observed in previous studies, CaO in LD slag is catalytical toward the WGS reaction during the conversion of solid fuel, affecting the reaction quotient *Q* during the gasification of the solid fuel.^13^ To see if the leached CaO of the weathered LD slag changes these properties, the reaction quotient *Q* was plotted against the conversion of solid fuel *X*_C_, as seen in Figure 10. Here, it can be seen that the difference between the fresh reference and the weathered LD slag material is present but small. However, at 970 °C, as seen in Figure 10, the difference between the reference and weathered sample is negligible for the interval 0.3 < *X*_C_ < 0.7. This suggests that it is the WGS reaction that is more efficiently catalyzed, promoting the reaction toward equilibrium affecting the gas composition ratio. This could be due to the increased surface area, more available CaO, and increased porosity that could be observed with decreased bulk density, as seen in Table 4.

## 4 Conclusions

In this study, the physical and chemical properties of LD slag were investigated regarding weathering from the aspect of using this material as an oxygen carrier on a large commercial scale. A sample was weathered by outdoor storage for 1.5 years in southwest Sweden before being analyzed. A part of the sample that was not agglomerated was used for experiments with both solid and gaseous fuels in a laboratory fluidized bed reactor to evaluate the changes in reactivity.

From this analysis, it could be observed that the reactivity of the LD slag was more or less unchanged after weathering, both regarding the conversion of gaseous fuels such as CO and also considering solid fuels. The characteristic catalysis of the water gas shift reaction was not affected by the weathering. After the weathering, the CaO/Ca(OH)_2_ ratio was unchanged in the bulk of the sample, disregarding that CaO had been leached during the weathering.

However, the physical properties of the weathered material were significantly degraded. First, the material partly agglomerated due to leached CaO solidifying as CaCO_3_. This suggests that it could be problematic to store the LD slag oxygen carrier outdoors. Second, the structural integrity of the particles was degraded due to the weathering, resulting in a higher attrition rate.

Overall, this study suggests that if LD slag should be used as an oxygen carrier in an industrial-scale facility, it needs to be stored in a dry silo. This is to prevent weathering after being treated to obtain the correct size fraction for the process and maintain the structural integrity of the particles.

## Supporting Information Available

Cycle with LD slag with a comparison to a sand reference for graphical support for calculations executed with eq E5; gasification experiments with the reference and weathered LD slag at 870 and 970 °C with gas concentrations; and calculated reaction rate wherefrom the points in Figure 9 are calculated (PDF)
