# Supplementary material for: Effect of Weathering on Steel Converter Slag Used as an Oxygen Carrier
Source: ACS Omega. 2023 Nov 30;8(50):47472–81. doi: 10.1021/acsomega.3c04051 (PMC10733948; doi:10.1021/acsomega.3c04051)
Supplement: Supplementary file 1 — ao3c04051_si_001.pdf [file ao3c04051_si_001.pdf]

Effect of weathering on steel converter slag used as an oxygen carrier

Fredrik HILDOR[1*], Henrik LEION[1] & Carl LINDERHOLM[2]

[1] *Chemistry and Chemical Engineering, Chalmers University of Technology, 412 93 Göteborg, Sweden*

[2] *Department of Space, Earth and Environment, Chalmers University of Technology, 412 96 Göteborg, Sweden*

[*]*Corresponding author, e-mail: fredrik.hildor@chalmers.se*

Abstract

Keywords: Steel converter slag, LD slag, Oxygen Carrier, Weathering

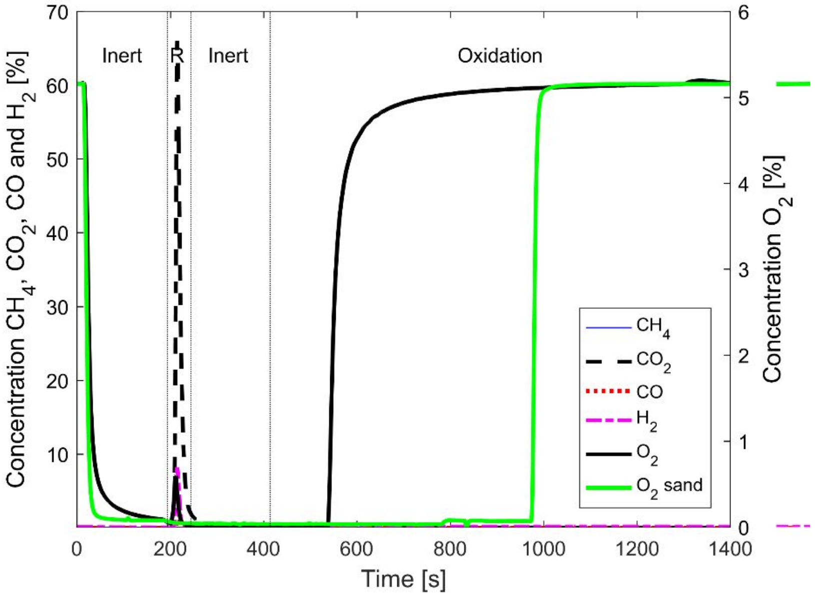

Figure S1. An activation cycle using LD slag as an oxygen carrier. A green line is plotted for the oxygen output in a reference experiment with sand. In the frirst "inert" period to the left difference between the green line and the black oxygen line is related to oxygen released by the oxygen carrier (CLOU – Chemical Looping with Oxygen Uncoupling).

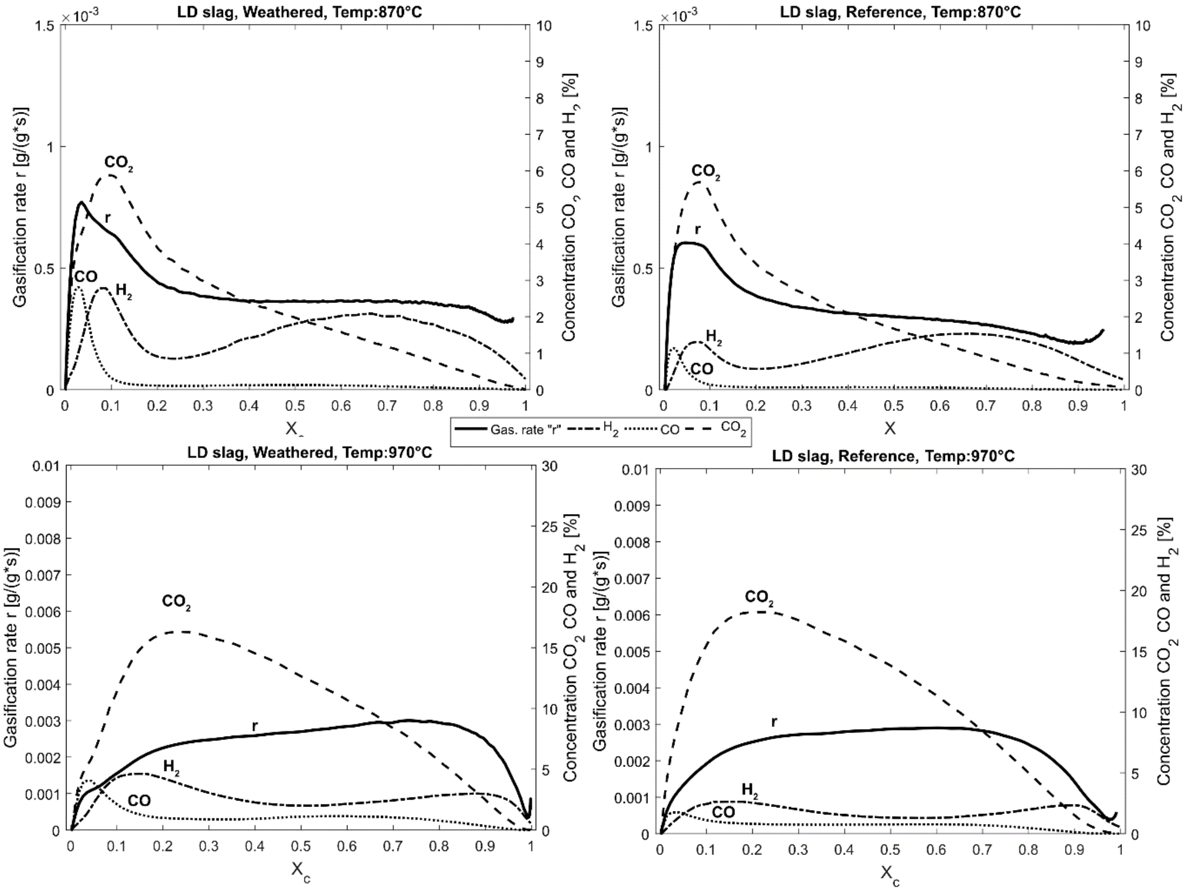

Figure S2. Gasification experiments with reference and weathered LD slag using german wood char as fuel at 870°C and 970°C. Here it can be observed some smaller differences between the weathered and reference experiments, but the overall shape is the same at both the different temperatures.
